# Photolysis of dimethoxynitrobenzyl-“caged” acids yields fluorescent products

**DOI:** 10.1038/s41598-019-49845-z

**Published:** 2019-09-17

**Authors:** Aleksey Yu. Vorob’ev, Tatyana Yu. Dranova, Alexander E. Moskalensky

**Affiliations:** 1 N.N. Vorozhtsov Novosibirsk Institute of Organic Chemistry SB RAS, 9 Lavrentiev Ave, 630090 Novosibirsk, Russia; 2 Novosibirsk State University, Pirogova 2, 630090 Novosibirsk, Russia; 3 Voevodsky Institute of Chemical Kinetics and Combustion SB RAS, Institutskaya str. 3, 630090 Novosibirsk, Russia

**Keywords:** Chemical modification, Single-molecule fluorescence, Biophotonics

## Abstract

Carboxylic acids conjugated with 4,5-dimethoxy-2-nitrobenzyl photoremovable protecting group are well known and widely used for biological studies. In this paper, we study the photolysis of likewise “caged” acetic, caprylic and arachidonic acids. Unexpectedly, we observed huge growth of fluorescence emission at ~430 nm during photolysis. Following further UV irradiation, a product with fluorescence at longer wavelength was formed (470 nm excitation / ~500–600 nm emission). While it may be used to monitor the “uncaging”, these fluorescent products may interfere with widespread dyes such as fluorescein in biomedical experiments. This effect might be negligible if the photolysis products dissolve in the medium. On the other hand, we observed that arachidonic and caprylic acids derivatives self-organize in emulsion droplets in water environment due to long lipophilic chains. Illumination of droplets by UV rapidly induces orange fluorescence excited by 488 nm light. This fluorescence turn-on was fast (~0.1 s) and apparently caused by the accumulation of water-insoluble fluorescent residuals inside droplets. These self-organized lipophilic structures with fluorescence turn-on capability may be of interest for biomedical and other application. We have identified and hypothesized some compounds which may be responsible for the observed fluorescense.

## Introduction

Light is used in different aspects of our life, from natural photosynthesis and to artificial photonic networks. All these processes rely on compounds that absorb light and transform its energy to the desired effect, including electromotive force^1–3^, chemical reactions^4^ and luminescence^5,6^. Application of photoremovable protecting groups to control the activity of biological molecules has been widely used and extensively studied^7–10^. It allows one to use light to “uncage” the molecule and rapidly induce the desired effect. For instance, lipo- or amphiphilic molecules conjugated with nitrobenzyl-based moieties have been used for preparation of photosensitive liposomes^11,12^. In such self-organized structures, light may be used to modify the properties of particles, for instance, destabilize the surface inducing cargo release. On the other hand, fatty acids are important messengers in biology, especially arachidonic acid. “Caged” derivatives of arachidonic acid were reported previously^13^, including that with water-soluble protective group^14^.

The ability of liquid hydrophobic substances to make emulsions in water is of great importance for life and technology. Emulsion contains dispersed phase consisting of small droplets or – in special cases – microdroplets, micelles or liposomes. Thus, water-insoluble molecules may be transferred and used in a water environment; a perfect example is dairy fat in milk and, more generally, fat digestion in a body. The dispersed phase may be used for delivery of chemicals, for instance, with diagnostic and therapeutics purposes. This possibility has attracted a lot of attention in recent years^15–18^. Stimuli-responsive particles like micelles or liposomes are especially promising since they allow the spatiotemporal control of their action^19^.

In the present work, we describe arachidonic and caprylic acids conjugated with 4,5-dimethoxy-2-nitrobenzyl-based protecting group (so called “dimethoxynitrobenzyl-caged” or “DMNB-caged”). We show that these compounds form emulsions in water (with small fraction of DMSO), in contrast to acetic acid modified in the same way. Apparently, photolysis products accumulate inside these self-organized lipophilic droplets. Surprisingly, we observed that the droplets become highly fluorescent after ~0.1 s illumination by UV LED.

The fluorescence of DMNB-“caged” compounds residuals after photolysis was observed in previous studies^20,21^, but it was attributed to the release of fluorescent target compound and used as a measure of “uncaging” degree. Our study shows that this approach could be a source of systematical errors. The majority of studies focus on the released (“uncaged”) molecule, while the properties of cleaved protective group are usually out of scope, with notable exception of^22^. Little is known of the primary photoproduct 2-nitroso-4,5-dimethoxybenzaldehyde and its subsequent derivatives. To our knowledge, all known properties are that it is potentially toxic^23^ and is considered non-fluorescent^13^, although quenches the fluorescence of coumarin 343^24^. In the paper, we confirm and study the fluorescence of the photoproducts, both in droplets and in a solution. It may be used to detect the “uncaging”, but also may interfere with widespread dyes such as fluorescein isothiocyanate (FITC) in biomedical experiments. On the other hand, the possibility to switch on fluorescence might be useful in imaging applications^25^.

## Results and Discussions

**Figure 1:**
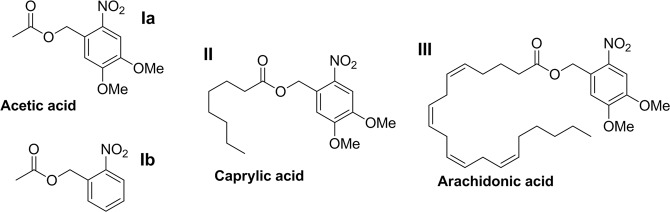
“Caged” compounds which were synthesized and used for experiments.

Figure 1 shows compounds **I**-**III** which we synthesized as described in supplementary materials and used in experiments. All compounds were dissolved in DMSO to the concentration of ~10 mM (stock solution).

**Figure 2:**
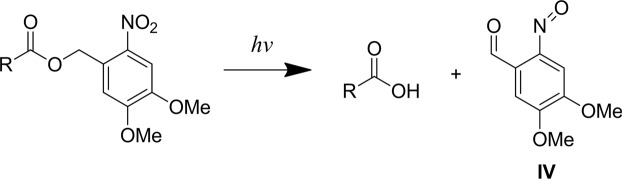
Presumable primary products of photolysis of **I-III** and analogous compounds: the “uncaged” carboxylic acid and 2-nitroso-4,5-dimethoxybenzaldehyde **IV**.

Photolysis mechanism of compounds conjugated with nitrobenzyl-based protecting groups has been described^26,27^. Although it may depend on the solvent and pH, generally two products are formed: the “uncaged” compound and the “byproduct”, or protecting group residual. The naive scheme for carboxylic acids is shown in Fig. 2, but, according to our results, it is only a part of the whole picture. Aromatic nitroso compounds could undergo photochemical transformations leading wide range of products^28,29^.

**Figure 3:**
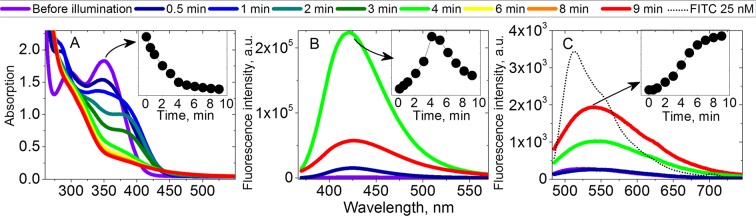
(**A**) Changes in an absorption spectra of “caged” acetic acid **Ia** in DMSO during UV illumination. (**B**) Fluorescence emission spectra of the same sample (excitation: 355 nm). (**C**) Fluorescence emission spectra of the same sample (excitation: 470 nm) and 25 nM of FITC for the comparison. Insets: time dependence of maximal value.

We first performed the “uncaging” experiment for 100 µM of **Ia** in DMSO. Figure 3A shows changes in absorption spectra during UV illumination as described in methods (365 nm, ~1 W). These changes almost stopped after 4–5 min., indicating the complete photolysis of the sample. We also measured fluorescence emission spectra upon 355 nm excitation wavelength and observed huge growth in fluorescence intensity following the UV illumination (Fig. 3B). The intensity at maximum (430 nm) has grown 150-fold after 4 min. of illumination. Interestingly, the growth was faster than linear, as shown in the inset, which means that formation of fluorescent product involves further reactions, e.g. dimerization (typical process for aromatic nitroso compounds like **IV**^30^**)**.

Further illumination caused the decrease of fluorescence at 430 nm. Absorption spectra did not change significantly during this process. The decrease was accompanied by the appearance of orange fluorescence excited by 470 nm light. Figure 3C shows the corresponding spectra and kinetics of the maximum value at 550 nm.

**Figure 4:**
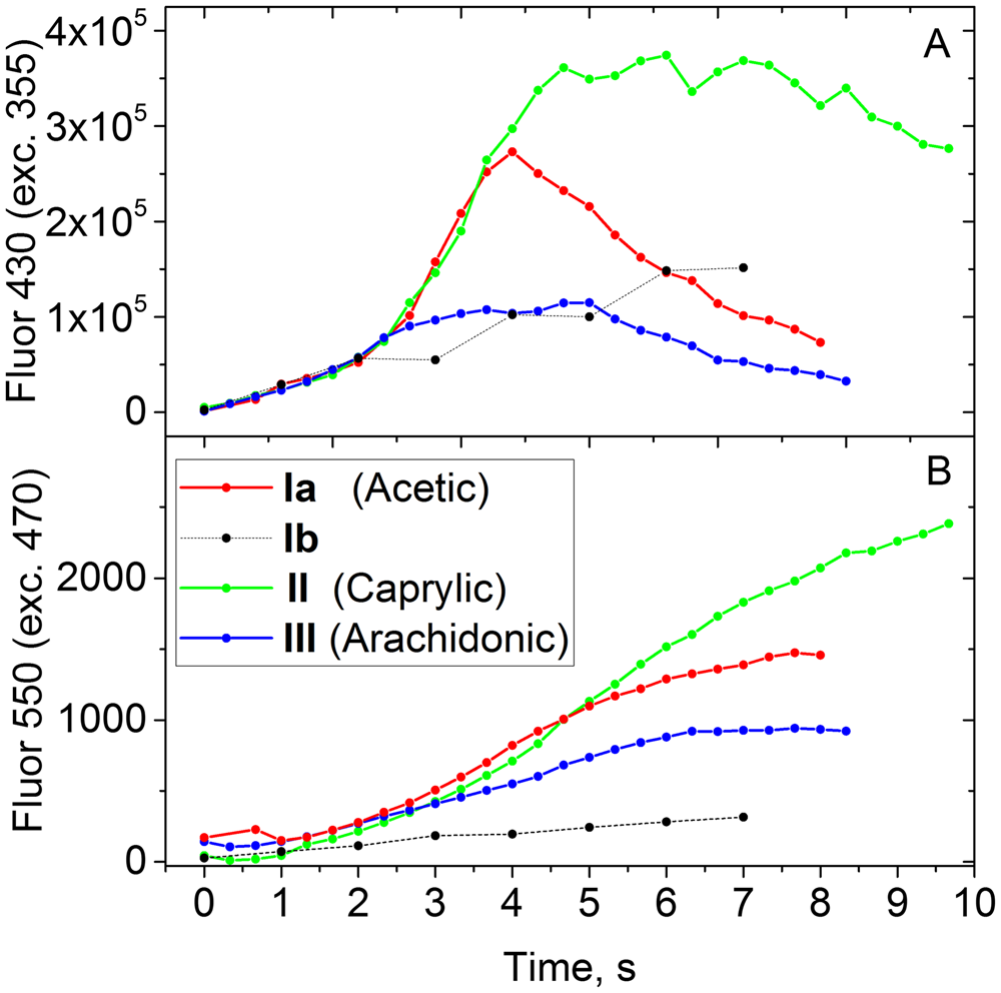
(**A**) Time dependence of fluorescence intensity at 430 nm (excitation: 355 nm) of **I-III** solution in DMSO following UV illumination. (**B**) Same samples, fluorescence excitation: 470 nm, emission 550 nm.

The fluorescence during photolysis of fatty acids **II** and **III** in DMSO follow the same pattern as **Ia**, although have different time course and maximal values (Fig. 4). Interestingly, the initial fluorescence growth is the same for all three compounds.

The kinetics of further decay of short-wavelength fluorescence and appearance of long-wavelength one depends on the acid used. It seems that free acid influences the ratio of different products, which the protective group forms after the photolysis. However, the normalized emission spectra of the ultimate product coincide exactly (Fig. S3). It shows that this fluorescent compound is likely the same for all three DMNB-based compounds. The compound thus should consist of several protective group residuals, either the same or different.

We can conclude that compound **IV** under our experimental conditions transforms into highly fluorescent derivative (355 excitation/430 emission). This derivative is subsequently converted by light to a form with longer-wavelength emission. Interestingly, the transition between two forms depends on concentration: in a 60-fold more concentrated solution the final form (470 nm excitation / ~500–600 nm emission) appear faster, with almost absent intermediate form (355 nm/430 nm). Fig. S2 shows the appearance of fluorescent product in a cuvette. To avoid possible artifacts and contamination, we reproduced this effect in several independent experiments, using both quartz and disposable plastic cuvettes, as well as a solvent from different sources.

Further, we tried to identify the fluorescent products. The *in situ* NMR study of reaction mixtures was performed simultaneously with fluorescence measurements using DMSO-d_6_ as a solvent and the only identified product was free acid (Fig. S1). The residual of protective group seems to form tens of different products with too little amount to identify by NMR. This was confirmed by GC-MS studies of reaction mixture after irradiation of compound **II**. However, we were able to identify 2-hydroxy-4,5-dimethoxybenzaldehyde and its *o*-caprilyc derivative (Fig. S5). Despite of these molecules has no data on fluorescence, their close analogs such as vanillin and 4-hydroxy-3,5-dimethoxybenzaldehyde has emission at 380–420 nm in polar solvents^31^, so we may hypothesize that these substances are responsible for shortwave emission. Presence of the acid residue in molecule with M_r_ = 308 explains the differences in graphs in Fig. 4. Indeed, the “uncaged” acid participates in chemical transformations of the protective group. Longer wavelength fluorescence may appear due to formation of dimeric products from 4,5-dimethoxy-2-nitrosobenzaldehyde under irradiation. Indeed, nitrozobenzenes are known to undergo photochemical dimerization with further transformations^29^ to form benzo[*c*]cinnoline derivatives, which has fluorescence maxima around 500 nm^32^ (Fig. S5). 3-Hydroxy-4,5-dimethoxybenzaldehyde also could be engaged in photochemical oxidative dimerisation similar to vanillin^33^. In GC-MS spectra, we observed substances with molecular weights of 356 and 359 which may correspond to such dimeric products. In previous studies^20,21^, the increase of fluorescence intensity was explained by the release of fluorescent target compound and was used as a measure of “uncaging” degree. Our study shows that this approach could be a source of systematical errors.

To test whether this effect is unique for the protective group with methoxy substituents, we synthesized compound **Ib**, which bears nitrobenzyl group. The photolysis was performed by LEDs with 340 nm peak wavelength. Interestingly, we observed similar growth of short-wavelength fluorescence with emission spectrum shifted to the left (Fig. S4). Thus, the short-wavelength fluorescent product retains some properties of the protective group, including the absorption region. It supports the idea that this fluorescence is a discharge of energy absorbed by a residual of protective group, which no longer can be spent on the dissociation. In this case the residual is 2-nitrosobenzaldehyde, which is known to be fluorescent^34^, or its derivatives.

Kinetics of fluorescence intensity at 430 nm is shown in Fig. 4 (dashed line). In contrast to DMNB-based compounds, there is no acceleration of growth for **Ib**. The formation of long-wavelength fluorescent product is almost absent. We can conclude that the reactions which yield that product are much more efficient and fast in case of DMNB protective group.

Usage in biological systems implies water environment, and we conducted separate experiments in phosphate buffered saline (pH 7.3). Stock solutions of **I**-**III** were gently mixed with ten-fold volume of PBS. For **II** and **III** we immediately observed characteristic milky appearance of mixture indicating the formation of emulsion. In contrast, acetic acid **Ia** dissolves well in the medium, which shows that self-organization into droplets occurs due to long lipophilic chains of fatty acids.

Light microscopy of emulsions showed round droplets with sizes in a range of 1–10 µm. We have not tried to obtain more narrow size distribution, although we expect that it can be easily done with more extensive mixing or using ultrasound. The droplets showed no fluorescence, but started to glow with orange color after 1-second pulse of 365 nm UV light without any background fluorescence.

**Figure 5:**
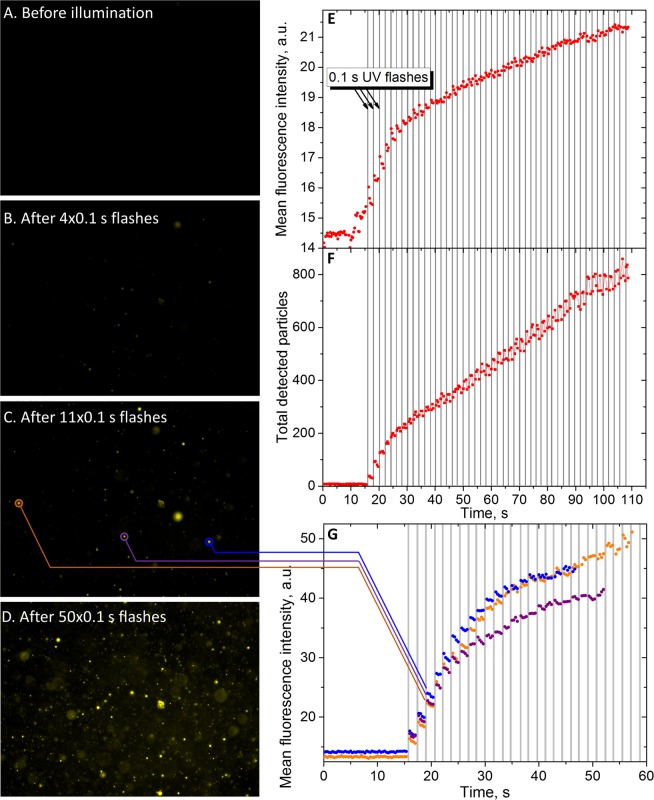
(**A**–**D**) Fluorescence images of emulsion (excitation: 450–490 nm; emission: >515 nm; 40x objective). (**E**) Mean fluorescence intensity of particles over time. (**F**) Total number of detectable particles over time. (**G**) Kinetics of fluorescence intensity for three single particles.

To study this phenomenon and relate it with our photolysis experiments in DMSO, we illuminated the droplets by 0.1 s UV flashes every 0.5 s and recorded the fluorescence intensity. Figure 5A–D shows images of droplets of **III** before illumination, after 4, 11 and 50 flashes. As particles were moving, we used the TrackMate FIJI plugin^35^ to measure the kinetics of fluorescence intensity for each particle. Figure 5E shows mean fluorescence value for all detected particles, Fig. 5F – the total number of detected particles over time and Fig. 5G shows kinetics of fluorescence for three single particles.

One can see that fluorescence started to grow immediately after the first flash, which indicates that all reaction stages are much faster in this case. We observed similar effect for compound **II**. The incredibly fast turn-on and the absence of background lead to the conclusion that photodecomposition product **IV** stays inside droplets after the photolysis, which is consistent with its water-insoluble structure. Its local concentration is much higher than that used in our photolysis experiments: it might be that of stock solution or even higher as the solvent (DMSO) may go outside the droplet. The fluorescence in droplets was stable for hours; after drying for 3 days, fluorescent fractal-like structure was formed, which is shown in Fig. S6.

## Conclusion

In conclusion, we have synthesized two “caged” fatty acids by conjugation with dimetoxynitrobenzol protecting group. The compounds showed efficient decomposition under UV light (365 nm). We also for the first time report fluorescent properties of the protecting group residuals after photolysis. Besides the fluorescence excited by ~355 nm, which is also present for usual nitrobenzol protecting group, DMNB version produces some compound whose fluorescence is significant under 488 nm excitation. This unknown compound would interfere with widespread dyes such as FITC and Green Fluorescent Protein in biomedical experiments, which should be kept in mind when using dimethoxynitrobenzyl protecting group.

Next, we showed that our fatty acids derivatives self-organize in emulsion droplets in water environment due to long lipophilic chains. Finally, the illumination of droplets by UV light induces fast fluorescence turn-on due to the accumulation of protecting group residuals inside. We have not identified the specific fluorescent compound, leaving it for the future research. However, such self-organized particles (droplets) may be of interest for biomedical and other applications^25^. For instance, a system where such particles are “turned on” by passing the laser beam may be used to trace hydrodynamic flows. More generally, transport of such particles from the particular place marked by a pulse of light can be studied in detail, including intracellular pathways.

## Methods

Synthesis of compounds **I**-**III** is described in supplementary materials.

The photolysis (“uncaging”) experiments were carried out in fluorimeter quartz cuvette with 10 × 10 mm light path. Compounds were dissolved in 3 mL of DMSO to the final concentration of 100 µM. We prepared a setup consisting of two UV LEDs with 365 nm central wavelength and 480 mW optical power. Another LEDs with peak wavelength of 340 nm were used in experiments with compound **Ib**. A microcontroller was used to set the illumination time, which was in a range from 0.1 s to 5 min in different experiments. After each session of illumination we measured UV-VIS absorption spectrum of the sample using Shimadzu UV-1900 spectrophotometer and fluorescence excitation/emission spectra with Shimadzu RF-6000 fluorimeter. Although we performed one measurement for each time point to avoid delays, all the described effects were reproduced in several independent experimental series.

Microscopic studies were conducted with Carl Zeiss AxioVert A1 with 450–490 excitation and >515 emission filters. All experiments were recorded using AxioCam 503 monochromatic high-sensitive camera with 3x analog gain and exposure time of 0.1 s. Illumination of samples was performed in place of observation with the same UV LED used in cuvette photolysis experiments. TrackMate FIJI plugin^35^ was used to measure the kinetics of fluorescence intensity for particles. We used LoG detector with the following parameters: estimated diameter 20 pixels, threshold 0.05.

NMR studies were performed on Brucker AV-500 spectrometer. DMSO-d6 was used as a solvent.

## Supplementary information

Supplementary information

## Data Availability

All the data obtained and used in the current study are available from the corresponding author on request.
