# Supplementary material for: Photolysis of dimethoxynitrobenzyl-“caged” acids yields fluorescent products
Source: Sci Rep. 2019 Sep 17;9:13421. doi: 10.1038/s41598-019-49845-z (PMC6748988; doi:10.1038/s41598-019-49845-z)
Supplement: Supplementary file 1 — Supplementary information [file 41598_2019_49845_MOESM1_ESM.pdf]

Aleksey Yu. Vorob'ev, Tatyana Yu. Dranova, Alexander E. Moskalensky

Photolysis of dimethoxynitrobenzyl-"caged" acids yields fluorescent products

Supplementary materials

Synthesis of caged acids

4,5-dimethoxy-2-nitrobenzyl acetate (Ia)

0.050 g (0.23 mmol) of (4,5-dimethoxy-2-nitrophenyl)methanol were dissolved in 5 ml of CH_2Cl_2. Then 0.1 ml of NEt3 were added followed with 0.1 ml of acetyl chloride. The mixture was stirred for 30 min at room temperature. After that, reaction mixture was transferred into separatory funnel and washed with 10 ml of H_2O. Organic layer was separated, dried over Na_2SO_4 and evaporated in *vacuo*. Solid residue was purified by column chromatography (SiO_2, CH_2Cl_2) to yield 0.044 g (74%) of 4,5-dimethoxy-2-nitrobenzyl acetate. ^{1}H-NMR (400 MHz, DMSO-d_6): 2.10 (3H, c); 3.87 (3H, c); 3.91 (3H, c); 5.36 (2H, s); 7.19 (1H, s); 7.69 (1H, s). Lit.: [1].

2-nitrobenzyl acetate (Ib)

Was obtained in the same manner as **Ia**. ^{1}H-NMR (400 MHz, $CDCl_3$): δ 2.14 (s, 3H), 5.50 (s, 2H), 7.45-7.70 (m, 3H), 8.11-8.15 (m, 1H). This data in accordance with [2].

4,5-dimethoxy-2-nitrobenzyl heptanoate (II)

0.288 g (1.3 mmol) of (4,5-dimethoxy-2-nitrophenyl)methanol were dissolved in 20 ml of CH_2Cl_2. To the solution, 0.110 ml of caprilyc acid were added and then 0.010 mg of DMAP. After that, solution of 149.8 mg of DCC (0.73 mmol) in 5 ml of CH_2Cl_2 was added dropwise with stirring. The mixture was stirred for 2 h and then evaporated. Solid residue was purified by column chromatography (SiO_2, EtOAc-hexane 1:3 *vv*) to yield 237 mg of 4,5-dimethoxy-2-nitrobenzyl heptanoate. ^{1}H-NMR (400 MHz, $CDCl_3$): 0.85 (3H, t, 7.0 Hz); 1.20-1.34 (m); 1.66 (2H, tt, 6.6 Hz, 6.5 Hz); 2.39 (2H, t, 7.6 Hz); 3.94 (3H, s); 3.96 (3H, s); 5.49 (2H, s); 6.98 (1H, s); 7.70 (1H, s).

4,5-dimethoxy-2-nitrobenzyl arachidonate (III)

Obtained similar to 4,5-dimethoxy-2-nitrobenzyl heptanoate starting from 0.108 g of arachidonic acid. ^{1}H-NMR (400 MHz, $CDCl_3$): 0.83 (3H, t, 6.9 Hz); 1.18-1.35 (6H, m); 1.72 (2H, tt, 7.6 Hz, 7.4 Hz); 2.00 (2H, q, 6.8 Hz); 2.09 (2H, q, 7.1 Hz); 2.39 (2H, t, 7.6 Hz); 2.72-2.81 (6H, m); 3.91 (3H, s); 3.93 (3H, s); 5.24-5.39 (8H, m); 5.46 (2H, s); 6.96 (1H, s); 7.67 (1H, s). Lit.: [3].

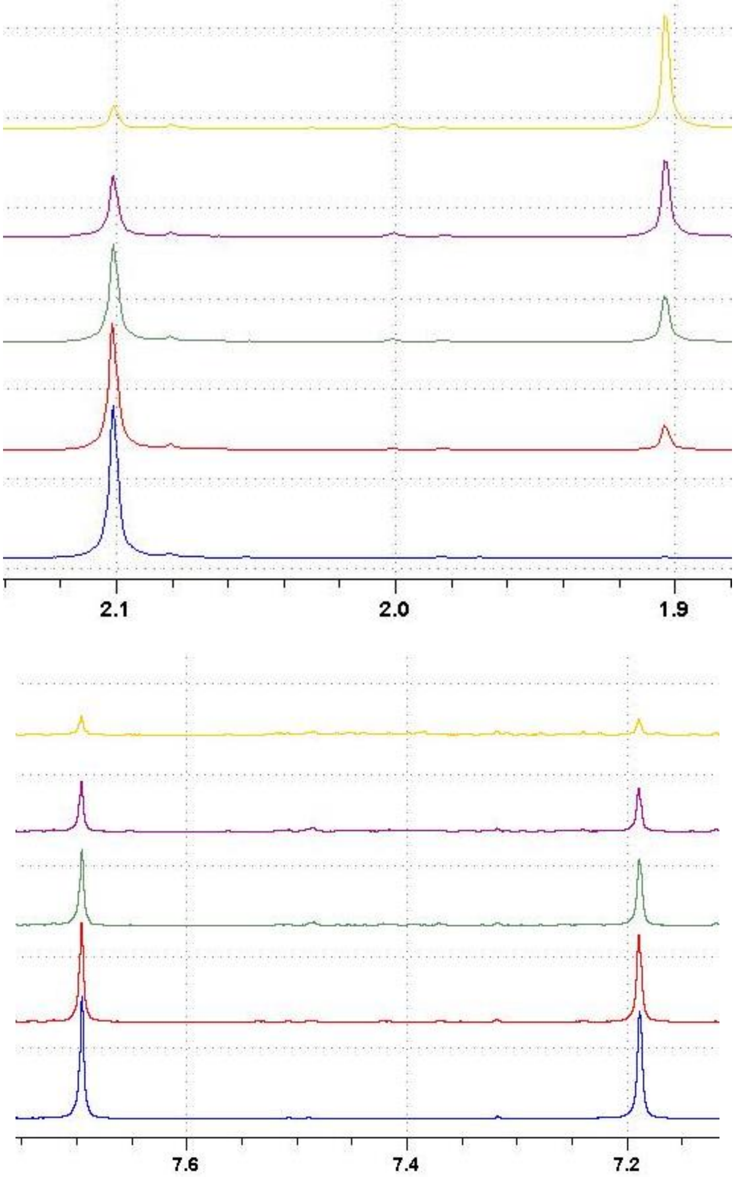

Fig. S1. ^{1}H-NMR of “caged” acetic acid (**I**, 10 mM in DMSO-d6) during photolysis (0 s, 30 s, 3.5 min, 6.5 min, 11.5 min). Upper panel: appearance of free acetic acid (1.9) and disappearance of conjugated one (2.1). Lower panel: disappearance of signals for the photoremovable protective group.

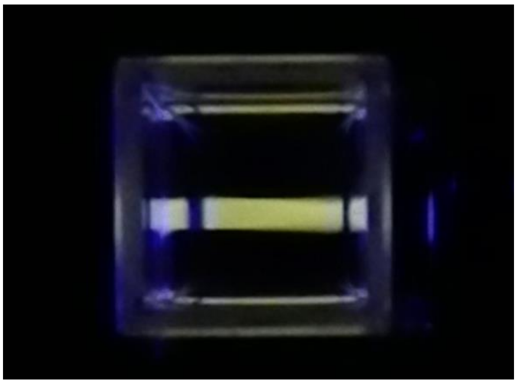

Fig. S2. Fluorescence in a 10 mm cuvette after several minutes of photolysis (upper view; excitation: 470 nm).

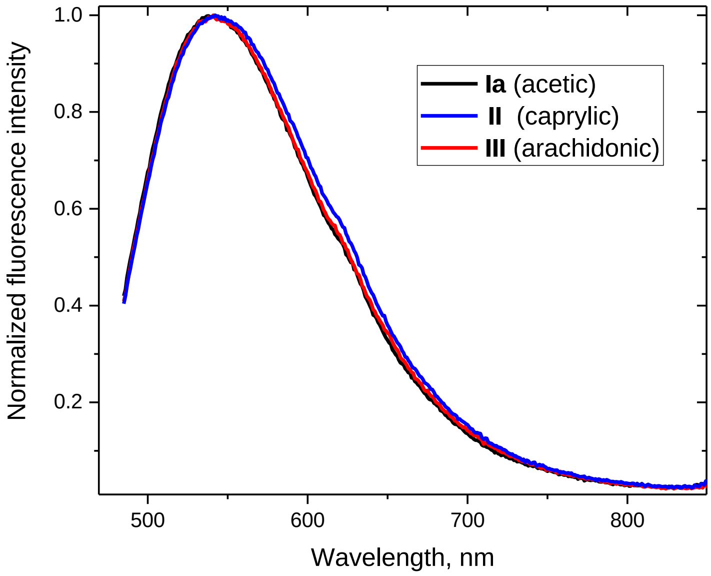

Fig. S3. Normalized “long-wavelength” fluorescence emission spectra after photolysis of **I-III**. Excitation 470 nm.

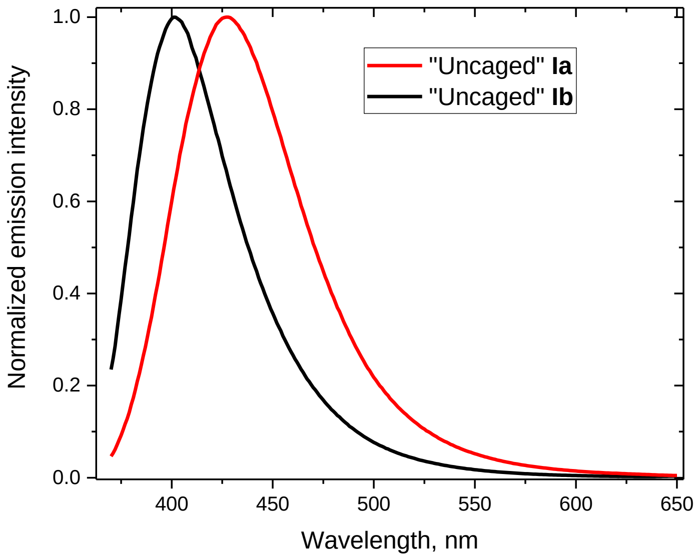

Fig. S4. Normalized fluorescence emission spectra after photolysis of **Ia** and **Ib**. Excitation 355 nm.

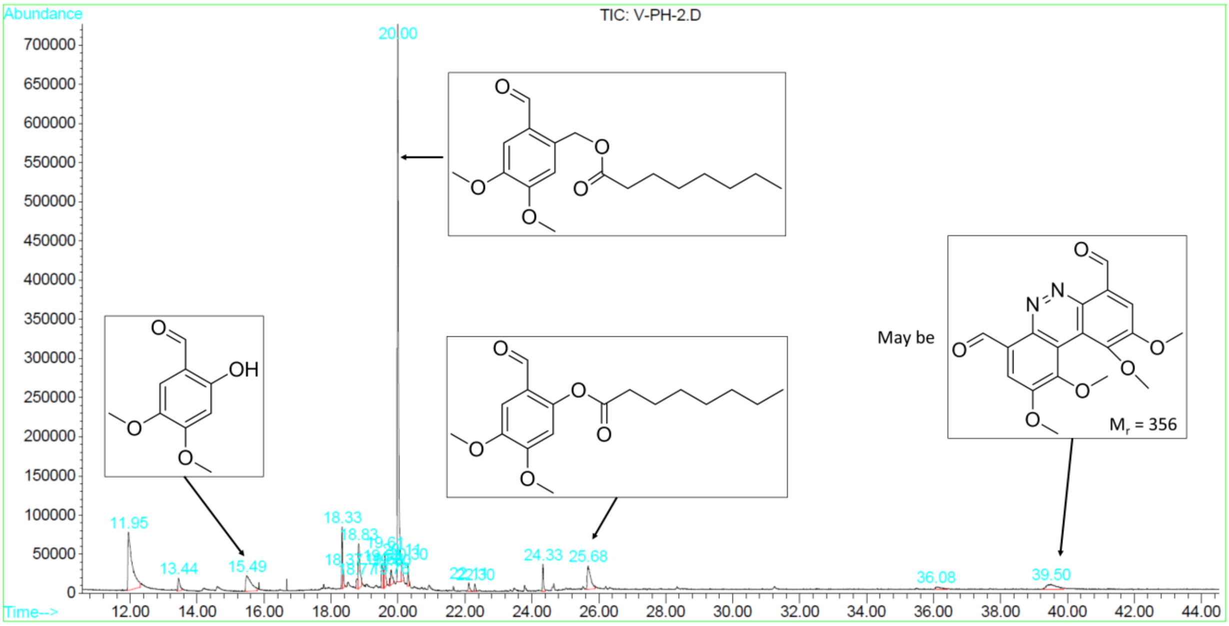

Peak 3 at Retention Time 15.49

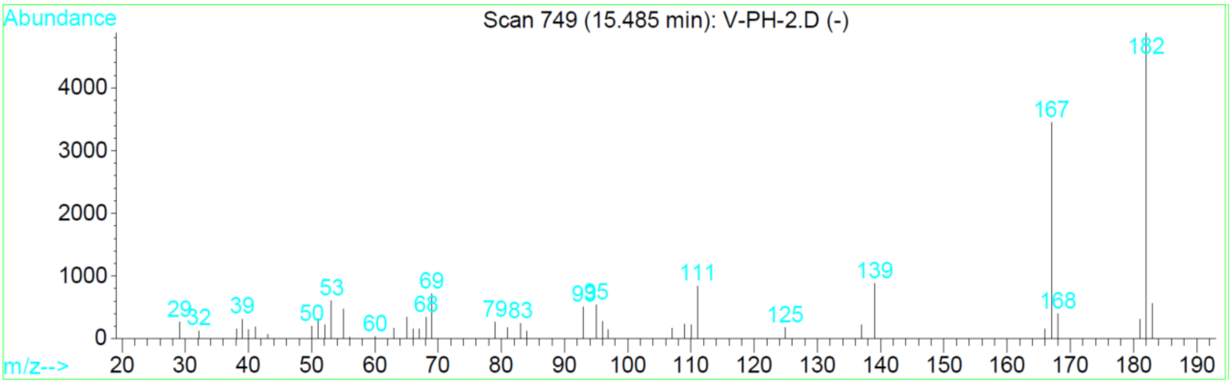

Peak 19 at Retention Time 25.68

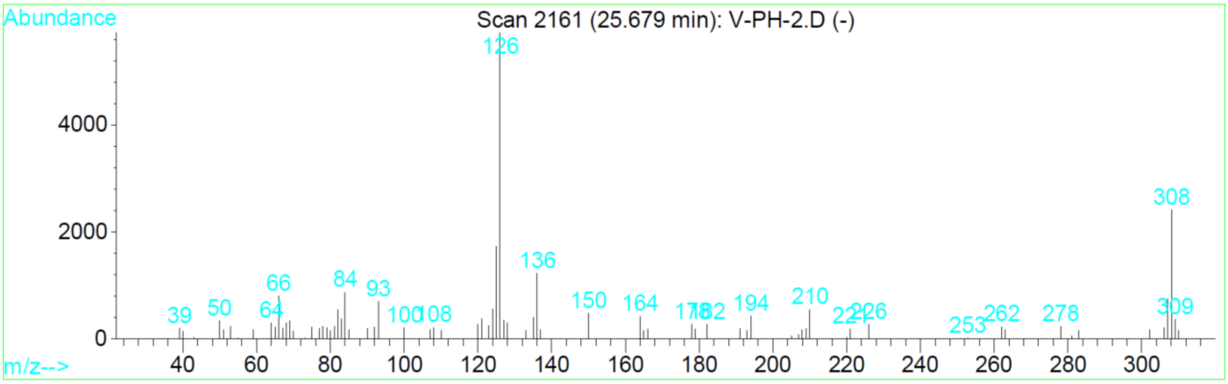

Peak 21 at Retention Time 39.50

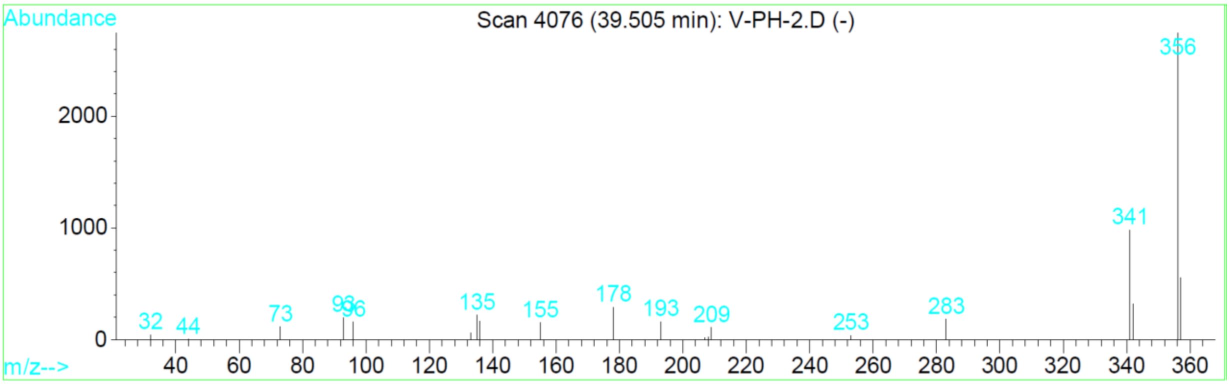

Fig S5. GC and mass-spectra of identified products for reaction mixture after compound 2 photolysis in DMSO.

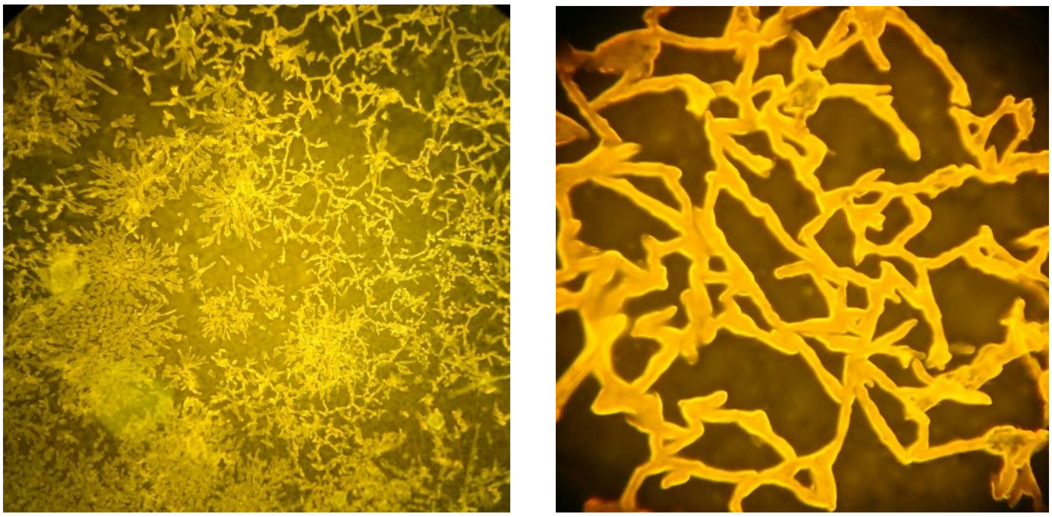

Fig. S6. Fluorescent product of decomposition of **III** in PBS + 10% DMSO after 3 days of drying in the air at room temperature. Left: 10x; right: 40x. Excitation: 450-490 nm.

References

[1] J. Lage Robles and C. G. Bochet, “Photochemical Release of Aldehydes from α-Acetoxy Nitroveratryl Ethers,” *Org. Lett.*, vol. 7, no. 16, pp. 3545–3547, Aug. 2005.

[2] G. Basumatary and G. Bez, “Ethyl acetate as an acetyl surrogate for the iodine catalyzed acetylation of alcohols,” *Tetrahedron Letters*, vol. 58, no. 45, pp. 4312–4315, Nov. 2017.

[3] A. Nadler *et al.*, “The Fatty Acid Composition of Diacylglycerols Determines Local Signaling Patterns,” *Angewandte Chemie International Edition*, vol. 52, no. 24, pp. 6330–6334, 2013.
